# Detection of human platelets antigen polymorphism (HPA-1 and HPA-3) and human factor XIII mutation in Sudanese women with recurrent pregnancy loss

**DOI:** 10.1186/s13104-024-06715-w

**Published:** 2024-03-05

**Authors:** Asaad MA. Babker, Huda Ahamed Fadlalmula, Amanda Gamal G. Elggourish, Hanan Khalid Fadul Ahmed, Shima Yousri Masoud Awad, Salaheldein G Elzaki, Rania Saad Suliman, Abdulkarim S. Bin Shaya, Abdulaziz Alfahed, Nahed S. Alharthi, Ahmed M Hjazi, Nora Y. Hakami, Mohammed Ageeli Hakami, Alhomidi Almotiri, Hisham Ali Waggiallah

**Affiliations:** 1 Department of Medical Laboratory Sciences, College of Health Sciences, Gulf Medical University, Ajman, United Arab Emirates; 2 Department of Epidemiology, Tropical Medicine Research Institute, National Center for Research, Khartoum, Sudan; 3 Department of Clinical Laboratory Sciences, Prince Sultan Military College for Health Sciences, Dhahran, Saudi Arabia; 4 Department of Medical Laboratory, College of Applied Medical Science , Prince Sattam Bin Abdulaziz University, 11942 Alkharj, Saudi Arabia; 5 Department of Medical Laboratory Sciences, Faculty of Applied Medical Sciences , King Abdulaziz University, Jeddah, Saudi Arabia; 6 Department of Clinical Laboratory Sciences, College of Applied Medical Sciences, Shaqra University, Al- Quwayiyah, Riyadh, Saudi Arabia; 7 Department of Clinical Laboratory Sciences, College of Applied Medical Sciences, Shaqra University, Dawadmi, Riyadh, Saudi Arabia

**Keywords:** Recurrent pregnancy loss, Human platelets antigen (HPA), HPA-1, HPA-3, Factor XIII

## Abstract

**Background:**

Recurrent pregnancy Loss (RPL) is common problem affecting many couples. A certain genetic variants link to increase the danger of this condition particularly *HPA-1, HPA-3* and Human Factor XIII Val34Leu Mutation. The present study aims to find an association between RPL and the Factor XIII Val34Leu polymorphism, as well as HPA-1 and HPA-3 in Sudanese women with RPL.

**Methods:**

This case-control study conducted between June 2022 and December 2022 included 216 women, with 103 cases having minimum three abortions in the past, and 113 healthy controls with at least two full-term births and no abortion history. DNA was isolated from whole blood and the status of three genetic polymorphisms (*HPA-1, HPA-3, and factor XIII*) was done using a polymerase chain reaction (PCR). Data was analysed using the SPSS version 24 software.

**Results:**

The A/A genotype was found to be more prevalent in cases (79.6%) and controls (96.5%) regarding HPA-1. A significant difference was observed in overall allele frequency for B allele (97.0%) and expected frequency of A allele was (81.1%) using the Hardy-Weinberg distribution (*p* < 0.001). The genotype A/A was most common in these patients (90.3%) and controls (100%), while B/B genotype was only (9.7%) in patients regarding HPA-3. Furthermore, the frequency of Val/Val genotype was higher in cases (88.3%) as compared with controls (90.3%). The risk of RPL in patients was nearly the same in Val/Leu individuals and controls group but all these differences were not statistically significant (*p* > 0.05).

**Conclusion:**

Our results indicate a link between Human Platelet Antigen-1 (HPA-1), Human Platelet Antigen-3 (HPA-3) and Factor XIII gene polymorphism with RPL.

## Introduction

Rrecurrent pregnancy loss (RPL) is a complex phenomenon characterized by two or more recurrent pregnancy miscarriages prior to the 20th week [1]. It has emerged as a reproductive health concern affecting 1–5% of pregnant women [2]. Recurrent miscarriage can have multiple etiologies, including endocrine, chromosomal, anatomical, genetic, and hereditary causes. Among the environmental causes, lead and ethylene oxide exposure are implicated to be potential causes of recurrent miscarriage [3]. Additionally, RPL may result due to immune system dysfunction and coagulation factor mutations [4]. Despite extensive investigations, approximately 40–50% of cases remain idiopathic [1]. Homeostasis is the physiological process that stops bleeding in damaged blood vessels. This process involves a series of interconnected steps that culminate in the formation of a “plug” that seals off the injured area. The first step in hemostasis is the constriction of the damaged blood vessel followed by the transient formation of a “platelet plug” which leads to activation of coagulation pathway, leading to the final clot or “fibrin plug” formation [5].

Hemostasis involves systemic activation of various enzymes that help in the process of clot formation using fibrin polymers and platelets [5]. This clot acts as a plug that controls bleeding and promotes tissue regeneration. As the wound begins to heal, the clot undergoes a process of remodeling, eventually dissolving as normal tissue growth replaces the damaged area [6].

Factor XIII, also known as fibrin-stabilizing factor, plays a crucial role in stabilizing blood clots during the coagulation cascade. It is a plasma protein heterodimer composed of A and B subunits, which are expressed by bone marrow and mesenchymal lineage cells. Factor XIII is a transglutaminase enzyme, catalyzing cross-linkage peptide reactions that contribute to the stability and strength of the fibrin clot [7]. As a member of the transglutaminase enzyme family, Factor XIII is an essential component of the blood coagulation system, and its activation is necessary for its transglutaminase function [8].

Deficiency in the enzymatic properties of transglutaminase, which is responsible for cross-linking the fibrin mesh, is a rare condition that can lead to life-threatening issues with clot stability and hemostasis [9]. Polymorphisms in human platelet alloantigen’s are also caused by substitutions of single base-pair causing replacement of amino acids. The human platelet antigen (HPA) system consists of over 12 biallelic antigen polymorphisms, with HPA-1 and HPA-3 being potential causes of neonatal alloimmune thrombocytopenia, post-transfusion purpura, and platelet refractoriness [10, 11].

Gene polymorphism in thrombophilia genes had been implicated with the risk of RPL. There are insignificant reports emphasizing the clinical relevance of genetic variants in *Human Platelet Antigen-1 (HPA-1), Human Platelet Antigen-3 (HPA-3) and Factor XIII* genes with the development of RPL, and no study until date had been reported on Sudanese women. The current study aimed to study the association between *human platelets antigen 1 and 3 (*HPA-1, *HPA-3) and Factor XIII Val34Leu* polymorphism with RPL.

### Subjects and methods

This case-control study was carried out at Sudan’s Omdurman Medical Hospital between June and December 2022. There were 216 women in total, with 103 cases having had at least three abortions in the past and 113 healthy controls having had at least two full-term births and no abortion history. The age was not matched between case and control groups visited the same hospitals due limited numbers. Women who had three or more consecutive RPL findings without being aware of the underlying cause met the inclusion criteria for the study. Due to limited resources only 103 available samples were included in this study, but the represent samples should be selected by following formula: n = N/1 + N(e)^2^.

n = sample size.

N = population of study.

e = margin error.

If a woman had a history of vascular thrombotic illness, congenital deformities, chromosomal abnormalities, uterine abnormalities, or an abortion for a known reason, she was disqualified from the study. Five ml of venous blood from each participant was extracted and put into a designated container after each had been interviewed.

### Molecular techniques

#### DNA extraction

Genomic deoxyribonucleic acid (DNA) can be efficiently and quickly extracted from up to 200 l of whole blood using GF-1 Blood DNA Extraction Kit (Selangor Darul Ehsan, Malaysia).

#### DNA quantification

The quality and quantity of DNA was checked by measuring the absorbance at 260 nm Gene Quant spectrophotometer (Amersham bioscence.UK).

### Genotyping

#### Factor XIII

**Fig. 1:**
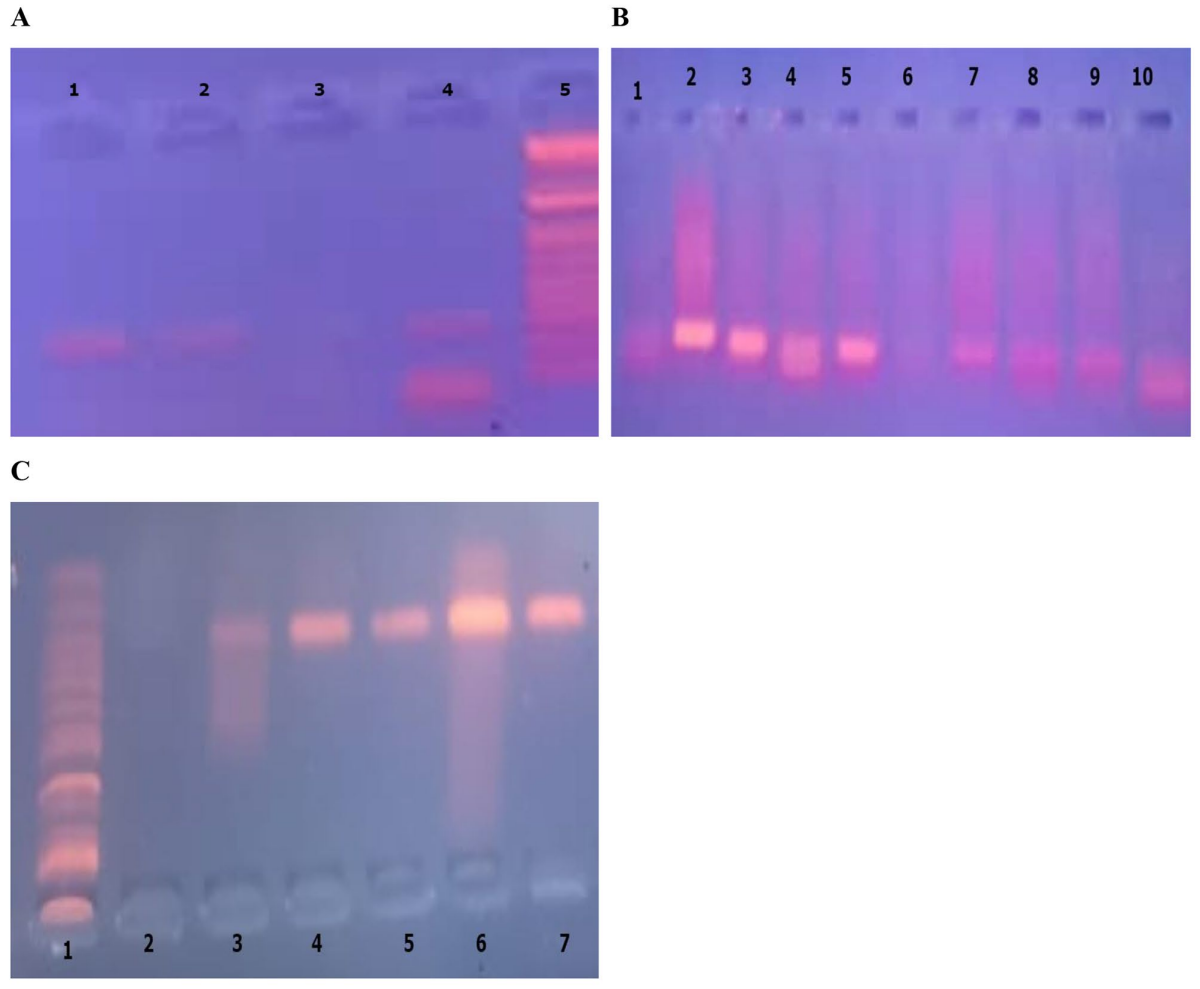
**A**. displays molecular genetic analysis of FXIII Val34 Leu polymorphism: Line 1 homozygous mutant (L/L) genotype, Line 2 homozygous mutant (V/V) genotype. Line 3 Negative control. Line 4 V/L heterozygous sample, Lane 5: 192 bp size marker. **B**: shows molecular genetic analysis of HPA-1: Polymerase chain reaction-restriction fragment length polymorphism (PCR-RFLP) products of HPA-1 gene variant; lin2 7, 8,9 mutant homozygote, 3,4,5 heterozygote, line 1,10: normal homozygote, line 6: negative control. Lane 11: 338 bp size marker. **C**: illustrates molecular genetic analysis of HPA-3: Lane 1: 253 bp size marker. Line 2 Negative A/B, Lines 3, 6 A/A. Lines 4,5,7,8 BB

To identify the *Val34Leu* polymorphism (Macrogen, Korea) (details mentioned in Table 1), also illustrates in (Fig. 1A).

#### HPA-1, and HPA-3

Genotyping was done using the polymerase chain reaction- Restriction Fragment Length Polymorphism (PCR-RFLP) technique; the T to C substitution at nucleotide 196 was investigated thermal cycler (Bio-RAD, USA) (UVP High-Performance UV Transilluminators Cambridge UK) as shown in Fig. 1 (B, C).

**Table 1:** Details of Primer sequences, PCR product size, Restriction enzyme and digested fragment size of studied gene polymorphisms

Gene	Primers sequences	PCR product size	Restriction Enzyme	Fragment size and Genotype
***HPA-1 (GpIIIa)***	F:5ʹCTGCAGGAGGTAGAGAGTCGCCATAG-3’	338 bp	Scarf	214/66/75 137/77/75/46 AA (Leu, Leu), AB (Leu, Pro) and BB (Pro, Pro)
	R: 5ʹ CTCCTCAGACCTCCACCTTGTGCTCT-3’			
***HPA-3 (GPIIb)***	F: 5ʹCTC AAG GTA AGA GCT GGG TGG AAG AAA GAC-3’	253 bp	FokI	253 AA 126/137 BB
	R: 5ʹCTC ACT ACG AGA ACG GGA TCC TGA AGC CTC-3’).			
***Factor XIII***	F: 5ʹCATGCCTTTTCTGTTGTCTTC-3’	192 bp	DdeI	192 Valine/Valine 161/3 Leu/Leu
	R: 5ʹ-3’ TACCTTGCAGGTTGACGCCCCGGGGCACTA			

### Ethical consideration

Each participant was interviewed, and verbal and written consent was obtained.

#### Ethical approval

was obtained from administration of the Omdurman Maternity Hospital, Khartoum, Sudan, (Approval code: OKH/OS/1A/77/1).

### Statistical analysis

The Chi square test was used to find the association of *HPA-1, HPA-3, and Factor IIIX* gene polymorphisms between cases and controls. *P*-values ≤ 0.05 were regarded as statistically significant. We used t-test for parametric data on continuous variables. We created a multivariate and univariate logistic regression analysis for determining coefficients and associated 95% CI. We also checked the genotype and allele frequencies of studied gene polymorphisms using Hardy-Weinberg equilibrium. These studies allowed us to draw significant statistical inferences. All the data was analyzed using SPSS software (version: 24.0) software.

## Results

We included 103 cases, and 113 control women subjects who met the eligibility criteria in this study. The average age of cases diagnosed with recurrent miscarriage 33.98 ± 5.57 as compared to controls (29.89 ± 5.45). About times of abortion, most cases (75.7%) had less than four times of abortion. The characteristics of the study group are mentioned in Table 2.

**Table 2:** Baseline characteristics of cases and controls

Characteristics	Cases (*N* = 103)	Controls (*N* = 113)	*P*-value
Age/ years (mean ± SD)	33.98 ± 5.57	29.89 ± 5.45	< 0.001
Times of abortion N (%)			
< 4	78 (75.7)	-	-
≥ 4	25 (24.3)	-	-

Data presented as n (%) Mean ± SD. Chi-square

*P-value < 0.01 very statistically significant association

**Table 3:** Association of *HPA-1, HPA-3* and *factor XIII* genotypes/alleles between recurrent miscarriage cases and controls

	103 Cases N(%)	113 Controls N (%)	Univariate		Multivariate	
			*P*-value	OR (95%CI)	*P*-value	OR (95%CI)
*HPA-1*						
A/A	82 (79.6)	109 (96.5)	-	1 (reference)	-	1 (reference)
A/B	3 (2.9)	3 (2.7)	0.34	1.33 (0.26–6.76)	0.608	1.53 (0.30–7.83)
B/B	18 (17.5)	1 (0.9)	0.002*	23.93 (3.13–1.82.90)	0.002*	24.13 (3.13-186.15)
Allele A	83 (80.6)	111 (98.2)	0.001*	10.3 (3.98–26.76)		
Allele B	20 (19.4)	2 (1.8)				
*HPA-3*						
A/A A/B	93 (90.3) 0 (0)	113 (100) 0 (0)	0.001* -	- -	- -	- -
B/B	10 (9.7)	0		-	-	-
Allele A	93 (90.3)	113 (100)	0.001*	-	-	-
Allele B	10 (9.7)	0		-	-	-
*Factor XIII*						
Val/Val	91 (88.3)	102 (90.3)	-	1(reference)	-	1(reference)
Val/Leu	10 (9.7)	9 (8.0)	0.649	1.25 (0.48–3.20)	0.530	1.38 (0.51–3.73)
Leu/Leu	2 (1.9)	2 (1.8)	0.910	1.12 (0.15–8.12)	0.673	1.53 (0.21–11.16)

Data presented as n (%) Mean ± SD. Chi-square. OR = odds ratio, CI = Confident Interval, - means not applicable due to absence the gene from control samples, *P-value < 0.01 very statistically significant association

In *HPA* gene, A/A genotype was most reported in cases (79.6%) and controls (96.5%). No significant difference was observed in A/B genotype frequency among cases and controls (*p* > 0.05). The frequency of B/B genotype was significantly higher in cases (17.5%) as compared to controls (0.9%), (*p* < 0.05) The overall allele frequency for the B allele was (97.0%), while the expected frequency of A allele was (81.1%) using the Hardy-Weinberg distribution and this difference was highly statistically significant (*p* < 0.001). Moreover, the *HPA-3* was identified in cases and controls as shown in Table 2. Its genotype A/A was most common in cases (90.3%) as compared to controls (100%), while B/B genotype was only (9.7%) in cases. This difference was statistically significant (*p* < 0.05). 90.3% of allele A and 9.7% of allele B were found in cases and this difference was highly statistically significant (*p* < 0.001). Furthermore, the Val/Val was most common in cases (88.3%) and controls (90.3%). The risk of miscarriage in patients was nearly the same in Val/Leu individuals and controls group and no significant differences were observed (*p* > 0.05).

In multivariate analysis, also, the *HPA-1* genotypes were carried out based on cases and controls. Most of these patients had higher B/B genotype compared to control with (*p* < 0.01; OR (95%CI): 24.13(3.13-186.15), Table 3.

**Table 4:** Distribution of time of abortion by *HPA-1, HPA-3* and *factor XIII* genotypes among cases

	Abortion times		chi^2^	*P*-value
	< 4	≥ 4		
HPA-1				
A/A	61 (78.2)	21 (84.0)	4.71	0.095
A/B	1 (1.3)	2 (8.0)		
B/B	16 (20.5)	2 (8.0)		
HPA-3				
A/A	72 (92.3)	21 (84.0)	1.49	0.222
B/B	6 (7.7)	4 (16.0)		
XIII				
val/val	69 (88.5)	22 (88.0)	0.82	
val/leu	7 (9.0)	3 (12.0)	0.664	
leu/leu	2 (2.6)	0		

Data presented as n (%) Mean ± SD. Chi-square and P value.

*P-value < 0.01 very statistically significant association.

The results of the study demonstrated the relation between *HPA-1and HPA-3* genotypes and times of abortions. In *HPA-1* genotype, 78.2% of the A/A genotype had less than four times and 84.0% had more or equal to 4 times. On the other hand, the *HPA-3* genotype A/A was most common (92.3%) with less than four times compared to those who had more or equal to 4 times. No association was revealed between *HPA-1and HPA-3* genotypes and times of abortion (*p* > 0.05). The same can be said to *factor XIII* where no significant association was observed. (*p* > 0.05) (details mentioned in Table 4).

**Fig. 2:**
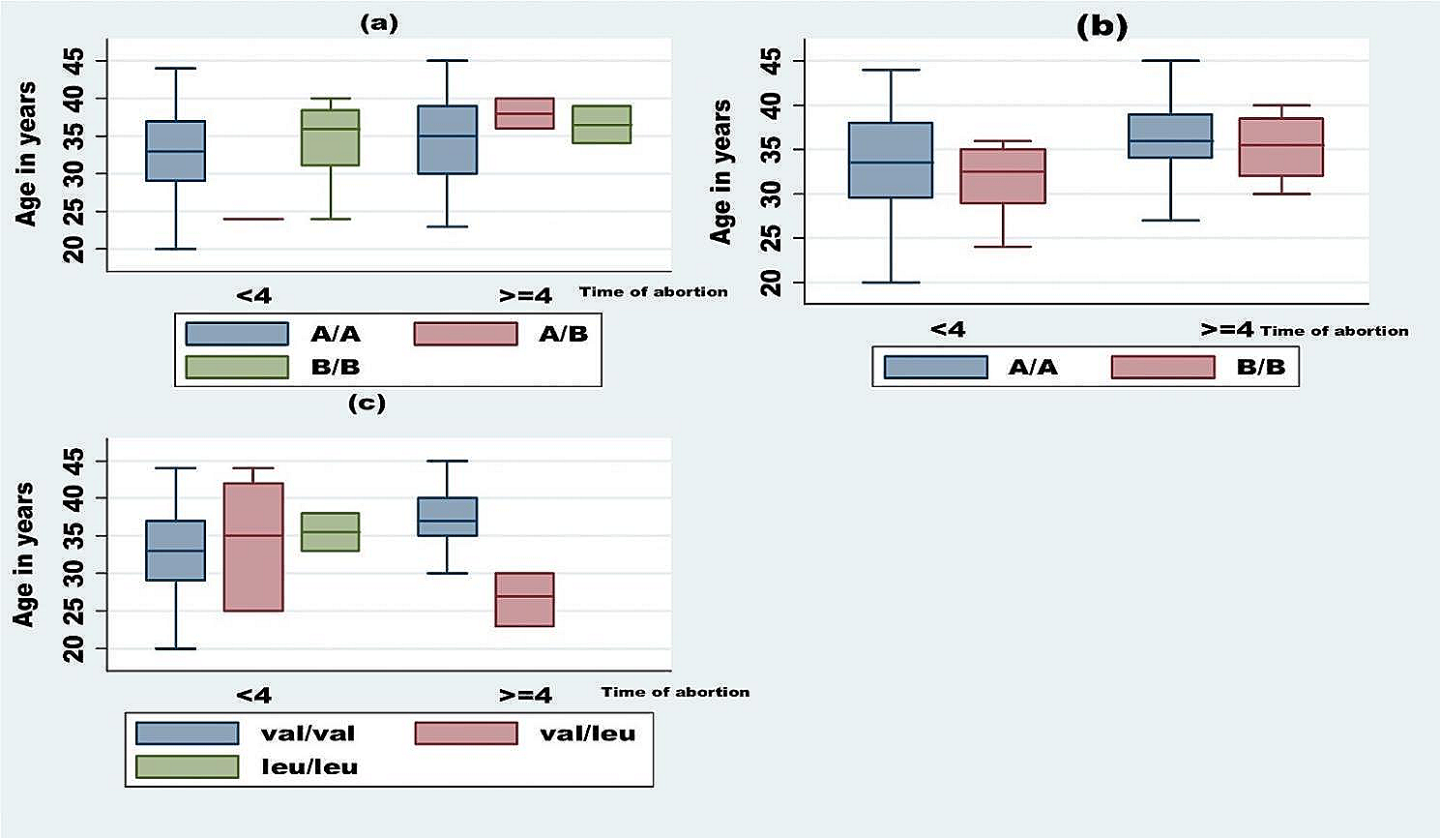
Box plot showing the distribution *HPA-1, HPA-3 and factor XIII genotypes* with age and time of abortion

The study group showed that there is a clear overlap between age and HPA-1 genotypes among abortion times. The age of patients according to the three main genotypes of HPA-1 was not different between times of abortion (*P*-value > 0.05), Fig. 2(a). In Fig. 2(b), the study observed the same overlap between the median age and HPA-3 genotypes among times of abortion. There is, also, an overlap between age of patients and factor XIII with times of abortion, Fig. 2(c).

## Discussion

In the current study the A/A genotype was found to be more prevalent in cases (79.6%) and controls (96.5%) regarding HPA-1. A significant difference was observed in overall allele frequency for B allele (97.0%) and expected frequency of A allele was (81.1%) using the Hardy-Weinberg distribution (*p* < 0.001). The genotype A/A was most common in these patients (90.3%) and controls (100%), while B/B genotype was only (9.7%) in patients regarding HPA-3. Furthermore, the frequency of Val/Val genotype was higher in cases (88.3%) as compared with controls (90.3%). The risk of RPL in patients was nearly the same in val/leu individuals and controls group but all these differences were not statistically significant (*p* > 0.05).

The Val34Leu variant is one of the most reported polymorphisms in factor XIII that is associated with various diseases including thrombosis, myocardial infarction, and cerebral hemorrhage [12, 13]. It had been extensively studied outside of Iran where it was found to be associated with risk factor for RPL; however, many reports did not find any association in Iranian Azeri women [14]. It has been concentrated on Val34Leu polymorphism in Factor XIII gene it is reported to be significantly associated with pregnancy outcomes.

Elmahgoub et al. hypothesised that *Factor XIII Val34Leu* polymorphism is associated with RPL and women carrying Val/Leu and Leu/Leu genotype of *factor XIII* are at higher risk of RPL events [15].

According to Li et al. FXIII Val34Leu polymorphism might significantly lower the incidence of RPL in both the dominant and co-dominant models (Val/Val vs. Val/Leu) [16].

However, Torabi and his co-worker. Did not find any association between of *IG103T* polymorphism, with RPL Iranian women [17]. Similary a study on Urmian population did not show any statistically significant results [18]. The findings of the current study concurred with those of investigations by Torabi [17] and Bagheri [18] where, Val34Leu polymorphisms therefore did not impair normal pregnancy and did not associate with pathophysiology of RPL in Irani women of Azeri descent.

The reason for these differences in different studies could be due to slightly different inclusion criteria’s, clinical differences, regional differences, insufficient sample sizes even on the same population.

According to our findings, there is no link between HPA-1, and HPA-3. However, Weiss et al. discovered that the patients with CVD-stroke and the inpatient control group revealed a higher prevalence of HPA-1b. Given that the allele frequencies of HPA-1b in all three groups studied are within the range of previously reported frequencies of HPA-1b in white populations, this change was not statistically significant. The frequency of the HPA-1b allele was much more prevalent (50%) in a sample of 42 patients under the age of 60 compared to the control group (13.9%) [19].

In a prospective cohort study, conducted by Ridker, found the HPA-1 polymorphism in a subset of 704 males with symptoms of myocardial infarction, stroke, or venous thrombosis. They found no evidence that having the HPA-1b allele increased one’s risk of myocardial infarction, stroke, or venous thrombosis in the future [20].

There is currently no justification for why the HPA-1b polymorphism of GPIIb/IIIa should be a risk factor for the manifestation of arterial channel occlusions based on experimental data [21, 22]. Some investigation has shown that platelets with the HPA-1b phenotype should bind less fibrinogen when activated with ADP than platelets with the HPA-1a phenotype [23]. Our results concluded that indicate a link between Human Platelet Antigen-1 (HPA-1), Human Platelet Antigen-3 (HPA-3) and Factor XIII gene polymorphism with RPL.

When evaluating a new patient who has experienced two or more spontaneous losses, it is critical to obtain products of conception karyotype results from previous losses, if available, or tissues from those losses for subsequent genetic analysis. Parental genetic screening including Single Gene Defects, Musculoskeletal gene defects, Immunologic gene defects, and Thrombophilic gene defects (TGD); Among these, the majority of reports have addressed factor V Leiden, prothrombin gene promoter mutations, activated protein C resistance, and mutations in methylenetetrahydrofolate reductase, plasminogen activator inhibitor, thrombomodulin, and annexin A5 genes [24]. According to the findings of this study Human platelet antigen − 1 and Human platelet antigen − 3 ought to be included as prenatal genetic tests (TGD) for RPL women.

### Limitations

There were numerous challenges to the research, the first of which was to increase the quantity of samples to boost precision and accuracy in the research, and the second was to assess the gene HPA-2 and determine its association to RPL.

## Conclusion

The current study indicated a link between HPA-1, HPA-3 and Factor XIII gene polymorphism with RPL. Testing for the Platelets Antigen Polymorphism HPA-1 and HPA-3 must be included in the standard assessment for RPL patients.

## Data Availability

The datasets used and/or analyzed during the current study available from the corresponding author on reasonable request.

## Abbreviations

HPA-1: Human platelet antigen-1
HPA-3: Human platelet antigen-3
RBL: Recurrent pregnancy Loss
Val: Valyl
Leu: Leucyl
DNA: Deoxyribonucleic acid
PCR-RFLP: Polymerase chain reaction- Restriction Fragment Length Polymorphism
*GpIIIa*: Glycoprotein IIIa
*GPIIb*: Glycoprotein IIb
TGD: Thrombophilic gene defects
CVD: Cardiovascular disease
ADP: Adenosine diphosphate
HPA-2: Human platelet antigen-2
